# Ocular manifestations and diagnosis of tuberculosis involving the uvea: a case series

**DOI:** 10.1186/s40794-023-00205-w

**Published:** 2023-11-15

**Authors:** Jennifer KS Tsui, Stephanie Hiu Ling Poon, Nicholas Siu Kay Fung

**Affiliations:** Department of Ophthalmology, Li Ka Shing Faculty of Medicine, The University of Hong Kong, 301B Cyberport 4, 100 Cyberport Road, Pokfulam, Hong Kong SAR China

**Keywords:** Ocular tuberculosis, TB uveitis, Ocular uveitis, Mycobacterium tuberculosis, Extra-pulmonary TB

## Abstract

**Background:**

Ocular tuberculosis (TB) affects 1–2% of patients with TB, with TB uveitis being the most common. This series aims to look at different manifestations of tuberculosis associated uveitis and the different tests used to make a presumptive or definitive diagnosis.

**Methods:**

Patients diagnosed with TB related uveitis in Hong Kong SAR between 2017 and 2020 were reviewed. Demographics, clinical features, investigations and treatments of patients were collected.

**Results:**

Fifteen eyes in 10 patients with a mean age 57.30 ± 10.17 years were included. The ocular manifestations on presentation included anterior uveitis (50%), posterior uveitis (40%) and panuveitis (10%), where 70% of them were unilateral and 30% were bilaterally infected; on subsequent visits the manifestations further developed into posterior uveitis (40%), panuveitis (40%) and anterior uveitis (20%), where 50% of them were unilateral and 50% bilateral infected. Tuberculosis tests were positive in 5 out of 7 Mantoux tests, 4 out of 4 T-SPOT TB tests, 3 out of 4 QuantiFERON-TB gold tests, 1 out of 1 lymph node biopsy, 0 out of 9 chest x-rays, and no aqueous fluid polymerase chain reaction (PCR) was tested. Vision impairing complications were seen in 6 patients where retinal vasculitis was most commonly seen. With anti-TB treatment prescribed in 9 patients, side effects occurred in 5 patients, including ocular hypertension, disc swelling, and hepatitis.

**Conclusions:**

Ocular TB infections may manifest in various forms, and can involve different parts of the eye. Bilateral involvement of TB is commonly presented, and both eyes should be evaluated at every follow up. When TB is suspected in a patient, diagnostic confirmation requires multimodal investigations where a negative chest x-ray is not useful in ruling out ocular TB infections, especially in an endemic region like Hong Kong. In these patients, it is crucial to have a high index of suspicion for TB, even when they do not demonstrate classical respiratory signs and symptoms of TB.

## Background

Tuberculosis (TB) is a chronic and airborne transmissible disease caused by *Mycobacterium tuberculosis*, mostly seen in adults. World Health Organisation (WHO) estimated around 10.6 million people diagnosed with tuberculosis worldwide in 2021, and 1.4 million deaths in the same year [1]. Latent TB infection in a person is defined as an infected person with no clinical manifestation of active TB, yet there is persistent immune response to TB antigen stimulation. Latent TB infection is non-infectious and does not cause any signs and symptoms; but 5 to 10% of latent TB infected people will develop TB disease within the first two years of infection, and the risk factors include the impaired immune system, diabetes and smoking [2, 3]. Tuberculosis is a granulomatous infection mostly affecting the lungs but may also cause extrapulmonary infections including any part of the eye with or without systemic manifestation. Hence, ocular tuberculosis should not be excluded even if pulmonary tuberculosis is absent.

Ocular tuberculosis can manifest clinically in a myriad of different presentations. Primary ocular tuberculosis, where the ocular structure is the primary site of infection, can present as lid abscess, conjunctival infiltration, phlyctenulosis, scleritis and interstitial keratitis [4]. Secondary disease can affect the uveal tissue, retina, and optic nerve. This can occur either by haematogenous spread, or as a hypersensitivity response from systemic tuberculosis infections [5, 6]. TB-related uveitis can be anterior, intermediate, panuveitis or posterior, with the lattermost being the most common presentation of intraocular TB [7–9]. It is postulated that *Mycobacterium tuberculosis* favours highly oxygenated tissue, therefore often affects the choroid, where the blood flow rate is highest in the eye [10]. Posterior involvement of TB is a complex condition in its own right, which can present in different manners, with the most common being multifocal choroiditis [4]. Other less common forms of posterior involvement include serpiginous choroiditis, tubercles, sub-retinal abscess, and retinal vasculitis, yet occurrences of serpiginous choroiditis and retinal vasculitis are more common in ocular TB, especially in high incidence settings.

So far it is still challenging to diagnose ocular TB due to its multiple forms of manifestations and currently, diagnostic tests can be used to assist in the diagnosis of TB, including immunologic (Tuberculin skin test and interferon-γ releasing assay (IGRA)), radiologic (chest x-ray and computer tomography), bacteriologic (smear and culture), molecular (nucleic acid amplification tests) and histologic (histopathology) tests [11]. All of these procedures have their benefits and limitations (Table 1) and there is still no consensus on the perfect strategy for TB diagnosis. Among them, tuberculin skin test (TST), IGRA and chest x-ray (CXR) are the most common way to diagnose tuberculous infection. Tuberculin skin test, also called Purified Protein Derivative (PPD) skin test or Mantoux test, is the most common and regular test but it is an invasive test where tuberculin will be injected into the skin [11]. According to Centers for Disease Control and Prevention (CDC) of the United States, induration ≥ 15 mm in low-risk people, ≥ 10 mm in moderate risk people (e.g. people from endemic area) or ≥ 5 mm in high-risk individuals (e.g. TB contact history, immunocompromised patients and typical chest x-ray changes) are considered positive in Mantoux test [12]. However, TST positive also can be seen in the previous infection of *Mycobacterium tuberculosis*, previous vaccination of bacillus Calmette–Guerin (BCG) or non-tuberculous mycobacteria cross-reaction [12, 13]. The reasons for false-negative result include anergy, recent TB infection within the past 8 to 10 weeks and a very young age. IGRA (T-SPOT.TB and QuantiFERON-TB gold test) is a blood test, but less likely affected by prior BCG vaccination and non-tuberculous mycobacterial infection, hence both of them share a higher specificity comparing with TST [14]. CDC suggested that IGRAs are preferred in patients who had BCG vaccination, whereas TST is preferred for children younger than 5 years old [15]. Chest x-ray is considered a tool to provide information about the existence of active or healed pulmonary tuberculosis [7, 8]. It is therefore only useful if there exist pulmonary involvement of TB, or presence of granulomatous cavities, typically seen at the lung apices, formed from previous pulmonary TB [16]. Unlike TB infection, TB disease itself is detected by histology, molecular and bacteriologic tests, as TB infection cannot be picked up by Acid-Fast Bacilli (AFB) culture and smear due to its quiescent state within the granuloma.

**Table 1:** Comparison of the commonest TB infection diagnosis tests

	Tuberculin Skin Test	Interferon gamma releasing assay	Chest X Ray
Advantages	• Low cost • Wide availability	• Higher specificity than TST • No need follow-up	• Low cost • Wide availability
Disadvantages	• Invasive • Common to have false-positive and false-negative result • Not recommend for children • Need follow-up for assessment	• Invasive • Not recommend for children	• Radioactive • TB uveitis may present with normal CXR

TST, Tuberculin skin test, IGRA, interferon-γ releasing assay; CXR, chest x-ray

The treatment regime of TB uveitis is similar to systemic TB which uses a combination of tablets including rifampicin, isoniazid, pyrazinamide and ethambutol for first 2 months, and continues to use rifampicin and isoniazid for 4 months [4]. The treatment can be prolonged to 9 months or even more according to clinical response. Oral corticosteroids are suggested to treat ocular complications such as retinal vasculitis and cystoid macular oedema. Nevertheless, the common side effects and precautions of anti-TB drugs and steroid should be noted, including hepatotoxicity, neuropathy, and renal failure [17].

In this case series, we aim to evaluate the various clinical presentations of TB uveitis in an endemic population here in Hong Kong. We also demonstrate the use of different investigations for the presumptive diagnosis of TB uveitis in our sample.

## Methods

This case series is a retrospective cross-sectional study. of All cases of tuberculous uveitis between 2017 and 2020 were collected retrospectively from Grantham Hospital in Hong Kong. The presumptive diagnosis of TB uveitis was based on a clinical suspicion of TB uveitis followed by a tuberculin skin test (TST). Patients with a negative results or an induration less than 20 mm on TST were arranged to complete IGRA tests, either T-SPOT.TB test or QuantiFERON-TB gold test. Those who could not afford these less invasive self-financed tests in the private healthcare sector were arranged to have a lymph node biopsy which was free of charge in the public sector. Demographics, medical information including past history, clinical manifestations, complications, chest x-ray and microbiological investigations, treatment and prognosis were collected. Clinical manifestations of the eye were diagnosed based on clinical presentation, including findings on physical examination, slit lamp examination, fundi imaging, optical coherence tomography imaging of the eye. Clinical manifestations, complications, investigations and treatment were analysed respectively for each patient and fundi images of patients with notable ocular manifestations were captured and presented. This study was approved by the Institutional Review Board (IRB) of The University of Hong Kong / Hospital Authority Hong Kong West Cluster HKU/HA HKW (IRB ref. UW22-549).

## Results

### Demographics and past history

A total of 15 eyes of 10 tuberculous uveitis cases with a mean age 57.30 ± 10.17 years ranging from 39 to 71 years were identified between 2017 and 2020, of which 3 were men and 7 were women, 9 Chinese and 1 Indonesian were identified. 20% of the patients had past history of latent TB and 40% of the patients had a history of uveitis where 20% had bilateral panuveitis, 10% had unilateral anterior uveitis and 10% were serpiginous choroiditis. 60% of patients had systemic diseases including hypertension (30%), renal impairment (20%), acute myocardial infarction (10%), stroke (10%) and Sjogren’s syndrome (10%). All patients were not immunosuppressed and were negative in virus serology including Varicella-Zoster Virus (VZV), syphilis and Human Immunodeficiency Virus (HIV). All patients had no previous alcohol nor smoking history.

### Clinical manifestation

**Fig. 1:**
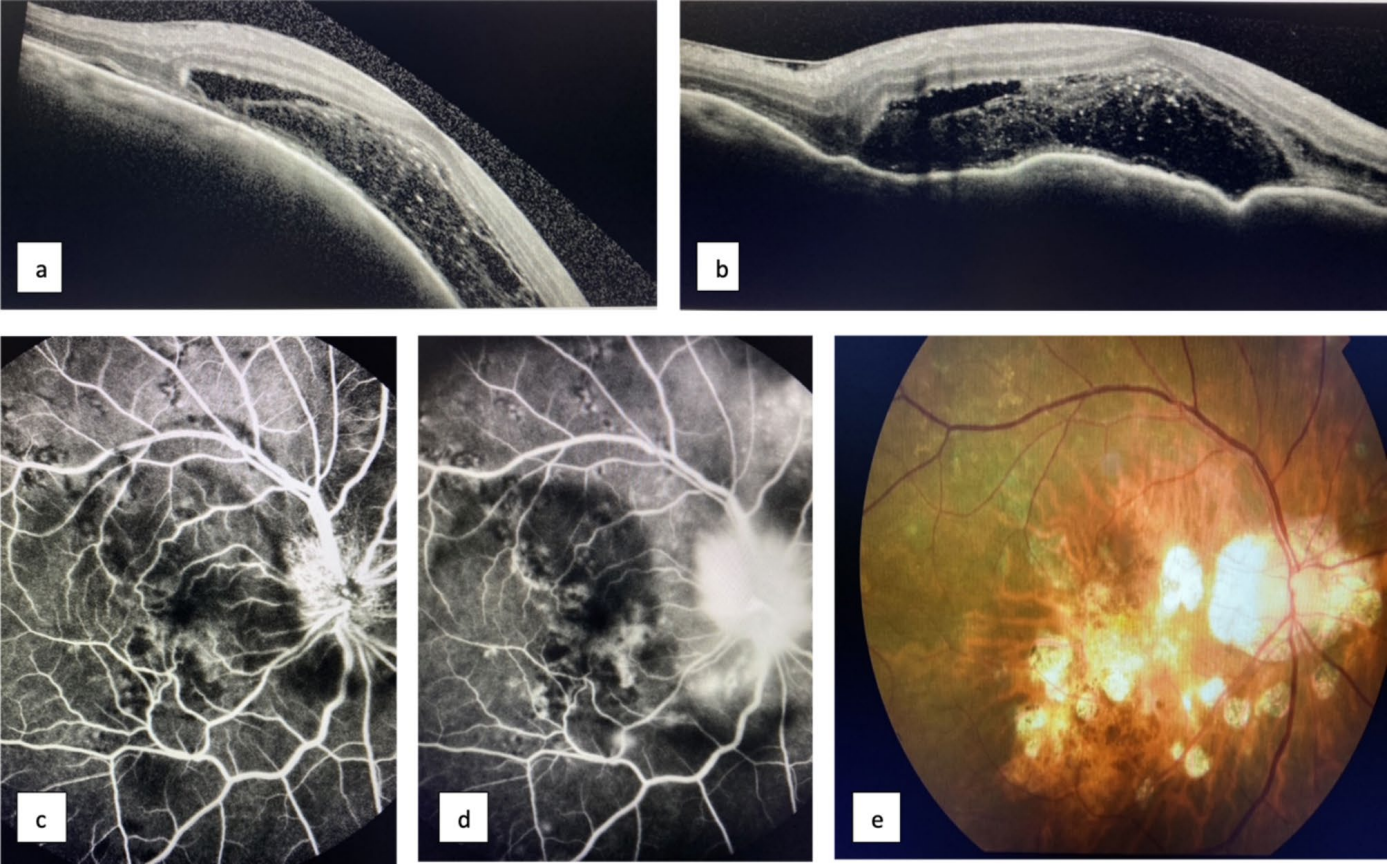
Ocular images of patient 7 with serpiginous choroiditis: (**a, b**) Optical Coherence Imaging showing sub-retinal exudates and fluid on presentation; (**c, d**) Fundus Fluorescein Angiography image showing disc leakage and non-perfusion area on presentation; (**e**) Fundus photo after infection was resolved

It is important to note that of all the patients in our case series, none presented with respiratory symptoms that could have pointed towards the diagnosis of TB. The most common presentation of uveitis in the first visit was anterior uveitis (50%), followed by posterior uveitis (40%) and panuveitis (10%). In the follow-up visit, two patients (patients 7 and 8) progressed to bilateral posterior uveitis from initially unilateral disease. One (patient 3) progressed from unilateral anterior uveitis to posterior uveitis of the same eye. Overall, three (patients 4, 5, 9) developed panuveitis on the second visit after presenting initially with either anterior or posterior uveitis on the same eye. Within these three, one (patient 5) developed vasculitis on the contralateral eye after presenting with anterior uveitis in that eye. With regards to laterality of involvement, on the first presentation, 70% of patients were unilaterally involved (4 in right eye and 3 in left eye), while 30% of patients were bilateral involved. In the follow-up visits, 50% of patients had unilateral infection and 50% had bilateral infection. The commonest signs were red eye (30%), painful eye (30%), blurry vision (30%), floaters (20%), photophobia (10%), reduced vision (10%), itchy eye (10%) and visual field defect (10%). The initial visual acuity was recorded on the first visit and the final visual acuity was recorded on the first visit following completion of the entire course of anti-TB treatment. 1 patient had extra-ocular manifestation showing progressive non-tender neck lymph node enlargement and excisional biopsy found granulomatous inflammation with acid-fast bacillus which was consistent with mycobacterial infection. No external infections nor systemic signs such as lymph node swelling, weight loss, night sweats, fever or cough was found in the remaining patients. 70% of patients developed ocular complications or side effects, including retinal vasculitis (40%), ocular hypertension (30%), cystoid macular oedema (20%), steroid dependence (10%) and TB drug-induced hepatitis (10%). Presented clinical features are summarised in Table 2. As shown in Fig. 1, disc leakage, non-perfusion area, sub-retinal exudates and fluid were observed.

**Table 2:** A summary of demographics and clinical manifestations

No.	Age	Sex	Previous history	Presenting complaint	Uveitis on presentation	Uveitis on follow-up visits	Initial VA of disease eye	Final VA of disease eye	Complications/ Side effects
1	39	F	No	Red & painful eye	Right Eye AU	Right Eye AU	+ 0.70	+ 0.15	Ocular hypertension
2	57	F	Past diagnosis of TB	Red & itchy eye	Right Eye AU	Right Eye AU	+ 0.52	+ 0.22	/
3	63	F	No	Floaters	Left Eye AU	Left Eye PU	+ 0.52	+ 1.00	Cystoid macular oedema
4	44	F	Contacted with TB meningitis patient	Red & painful eye, photophobia	Left Eye AU	Left Eye panuveitis	+ 0.70	+ 0.10	Disc swelling
5	48	M	Idiopathic panuveitis in 2013	Blurry vision	Both Eyes AU	Right Eye panuveitis; Left Eye vasculitis	+ 0.52/ +1.00	+ 0.70/ +1.00	Retinal vasculitis, ocular hypertension
6	71	F	No	Visual field defect	Right Eye PU	Right Eye PU (serpiginous choroiditis)	+ 0.30	+ 0.22	/
7	62	M	Serpignous choroidits in 2009	Reduced vision	Right Eye PU (serpiginous choroiditis)	Both Eyes PU (serpiginous choroiditis)	+ 2.30/ + 0.70	+ 1.80/ + 0.4	/
8	68	F	Idiopathic Left Eye AU in 2012	Floaters	Left Eye PU	Both Eyes PU	+ 0.70/ + 0.30	+ 0.40/ +0.40	Retinal vasculitis, cystoid macular oedema, ocular hypertension
9	55	F	Both Eyes panuveitis in 2013	Blurry vision	Both Eyes PU	Both Eyes panuveitis	+ 0.52/ +0.52	+ 1.30/ +1.00	Retinal vasculitis, steroid dependent
10	66	M	No	Painful eye	Both Eyes panuveitis	Both Eyes panuveitis	+ 1.53/ +1.70	+ 1.00/ + 1.80	Retinal vasculitis, TB drug-induced hepatitis

Visual Acuity is shown in LogMAR form. AU, anterior uveitis; F, female; Hx, history; IU, intermediate uveitis; M, male; N.A., not applicable; No., number; PU, posterior uveitis; VA, visual acuity

### Investigations

Tuberculosis tests were positive in 5 out of 7 TST, 4 out of 4 T-SPOT.TB test, 3 out of 4 QuantiFERON-TB gold test, positive in 1 out of 1 lymph node biopsy and 0 out of 9 chest x-ray. TST was performed in 7 patients; of these, 4 had 20 mm or greater induration diameter and the remained showing 17, 11 and 8 mm respectively. Patients with less than 20 mm or negative in TST were arranged to complete IGRA tests either T-SPOT.TB test or QuantiFERON-TB gold test and all of them were positive in IGRA tests. All other virus serology tests were negative. Apart from the lymph node involvement of TB in one patient, there were no extra-ocular TB involvement in the rest of the patients. A summary of the results of different TB tests is shown in Table 3.

**Table 3:** A summary of different TB diagnostic test results

Patient	Tuberculin skin test induration (threshold used)	T-SPOT.TB test	QuantiFERON-TB gold test	Chest x-ray	Neck lymph node biopsy
1	26mm (10mm)		-	-	
2	20mm (5mm)			-	
3	-		+		
4	8mm (5mm)	+		-	
5	-			-	+
6	20mm (10mm)	+		-	
7	11mm (10mm)		+	-	
8	17mm (10mm)		+	-	
9	20mm (10mm)	+		-	
10	-	+		-	

PCR, polymerase chain reaction; +, positive; -, negative

### Treatment and clinical outcome

Anti-TB treatment was prescribed and monitored by the TB Clinic (Sai Ying Pun Chest Clinic) and patients had ophthalmic follow-up visits in the eye clinic (Grantham Hospital). Anti-TB treatment was initiated in 9 patients and all of them completed a full course of anti-TB treatment for a total of 9 to 12 months and averaging 11.11 months, except 1 patient who refused to initiate anti-TB treatment. In terms of visual outcome, 53.5% of eyes had improved VA, 6.67% of eyes had no change in VA and 40% of eyes had worsen VA.

Topical steroids were prescribed in all 10 patients where 30% had ocular hypertension requiring an intraocular pressure (IOP) lowering agent (timolol) for 4 to 15 weeks. 1 patient developed steroid dependence. 1 patient who was on Cyclosporin A stopped the drug due to renal impairment. With regards to TB drug side effects, 1 patient was prescribed Isonizaid, Rifampicin, Pyrazinamide and Ethambutol as the initial treatment, and stopped ethambutol 2 months later due to disc swelling caused by it. In addition, 1 patient developed TB-drug induced hepatitis, requiring drug titration. Table 2 summarizes the demographics, clinical manifestations and treatment of patients with TB uveitis.

## Discussion

### Clinical manifestation

**Fig. 2:**
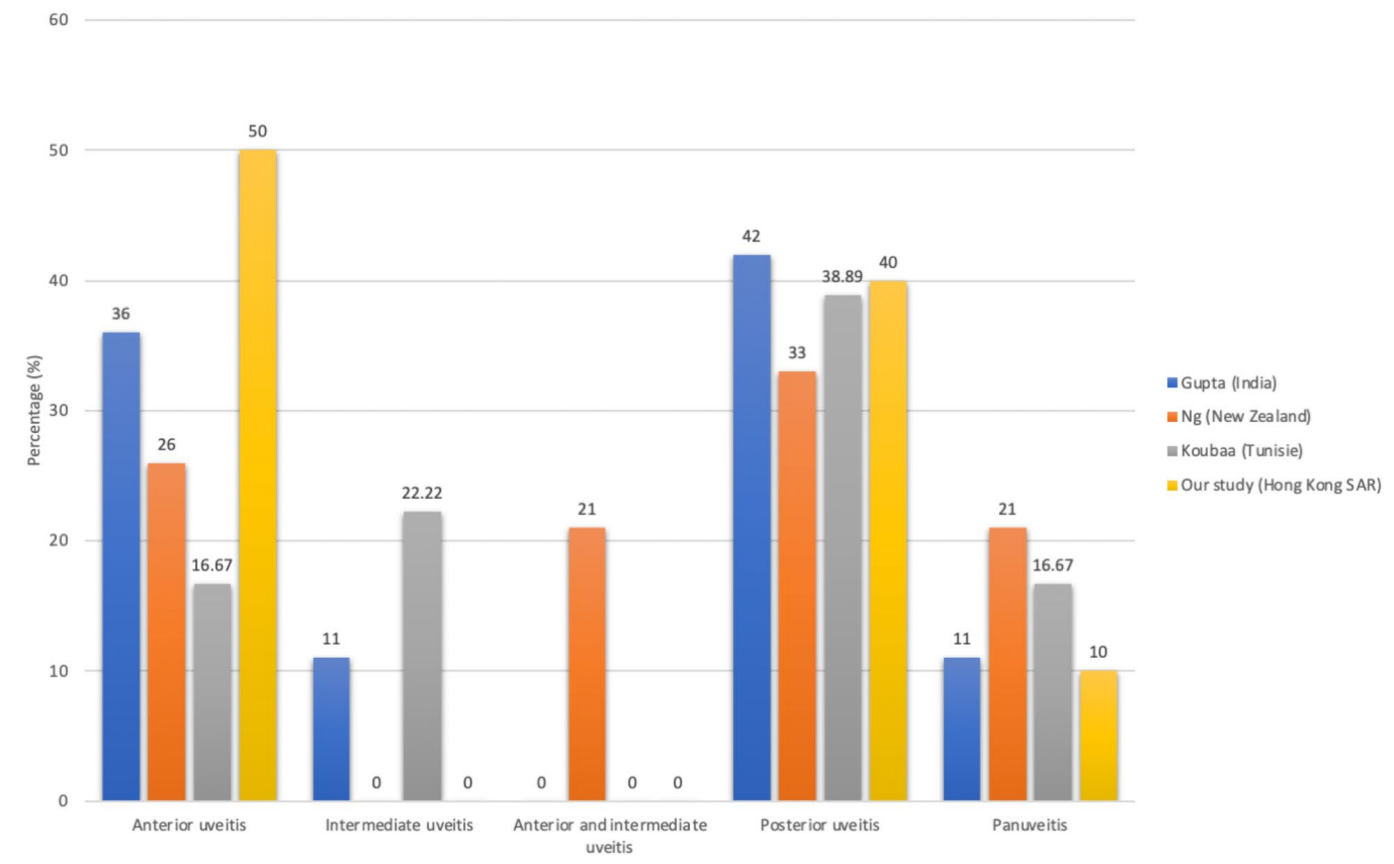
Comparison of the clinical spectrum TB uveitis at initial presentation among 4 studies (Referred to [7–9]). Anterior uveitis is defined as iritis, iridocyclitis, or anterior cyclitis, with the inflammation located predominantly at the anterior chamber. Intermediate uveitis is defined as pars planitis, posterior cyclitis, or hyalitis, with predominant inflammation at the vitreous. Posterior uveitis is defined as focal, multifocal, or diffuse choroiditis, chorioretinitis, retinochoroiditis, retinitis, neuroretinitis, with inflammation primarily at choroid or retina. Panuveitis is defined as inflammation of all three locations – anterior chamber, vitreous, and choroid or retina. [27]

Studies have shown that posterior uveitis is the commonest type of TB uveitis from Gupta (42%), Ng (33%) and Koubaa (38.89%). Our study found anterior uveitis (50%) to be the most common initial presentation while posterior uveitis (40%) and panuveitis (40%) are most commonly seen on follow-up visits. No intermediate uveitis was reported in our study. (Fig. 2)

In our cases series, 30% of patients first presented with bilateral infection. In terms of posterior uveitis, 1 out of 4 in the first presentation and 2 out of 4 in follow-up visit were classified as serpiginous-like choroiditis. Since different studies show different clinical spectrums, we suggest that monitoring of both eyes is essential as unilateral infection can develop into bilateral involvement, as 5 out of 10 patients subsequently presented with bilateral uveitis at follow up. The most common chief complaints were red eye (30%), painful eye (30%) and blurry vision (30%), while photophobia (10%), reduced vision (10%), itchy eye (10%) and visual field defect (10%) were less commonly seen.

In this study, 70% of patients developed disease associated complications or side effects showing retinal vasculitis (40%), cystoid macular oedema (20%), ocular hypertension (30%), disc swelling (10%), steroid dependent (10%) and TB drug-induced hepatitis. Among 6 patients with prior history, only 3 of them developed complications in our series. We found that 3 out of 4 patients who developed retinal vasculitis were diagnosed with panuveitis, hence we suggest that patients with panuveitis may be prone to develop complications, of which in our series, retinal vasculitis is a commonly seen complication in panuveitic patients.

### Diagnosis of TB uveitis

All patients in this case series were diagnosed with TB infection presumptively by at least one clinical sign together with a positive result of immunological or histological test. As the clinical signs and symptoms in TB uveitis are similar to other pathological causes of uveitis, TB diagnostic tests are mandatory to help the diagnosis of TB uveitis. In this study, only 20% of patients had prior known history of TB, which supports that ocular TB involvement cannot be ruled out in patients without history of systemic TB, especially in TB endemic regions. This also holds true for evaluating patients with previous presentation of idiopathic uveitis, for which the cause of the previous uveitic episode may not have been fully worked up for TB, or that the tests showed a false negative for the sample taken at the time. It is important that we think of TB as a differential diagnosis when encountering patients with uveitis in endemic areas, even when these patients do not present with the classical respiratory symptoms of TB, as TB is an entirely treatable and reversible cause of uveitis. In our case series, we demonstrated 40% of patients having had previous idiopathic uveal infection. Other aetiologies of uveitis should also be ruled out, such as herpes simplex virus (HSV), Epstein Barr virus (EBV), cytomegalovirus (CMV) and varicella zoster virus (VZV). With respect to the investigation of TB uveitis, chest x-ray and five types of TB tests including Tuberculin skin test, T-SPOT.TB test, QuantiFERON-TB gold test, and neck lymph node biopsy were used in different patients.

The least invasive investigative tools are usually the most acceptable to patients. However, this needs to be balanced with the diagnostic power of the respective tests. Chest x ray imaging, although regarded as one of the least invasive out of all the aforementioned investigations, provides the least diagnostic power for TB uveitis. There are several reasons as to why this is the case. Firstly, chest x ray can only detect pulmonary involvement of TB, including active pulmonary TB infection, or old TB with granulomatous cavitation which are more commonly seen at the lung apices. In addition, lesions seen at the lungs may not be entirely specific for TB, since there could be a wide variety of mimickers including lung abscess, enlarged mediastinal lymph nodes, lung carcinoma, secondary lung metastasis, or other mediastinal tumours including germ cell tumours which could also present similarly with lung nodule, sometimes also with cavitation, and pleural effusion and parenchymal opacities [18]. Further investigations must be performed to rule out other differential diagnoses of pulmonary lesions including the aforementioned. Similarly, other non-invasive examination tools including slit lamp, fundoscopy and optical coherence tomography (OCT) merely demonstrate active involvement of the ocular structures, yet fail to demonstrate the nature of the pathology, nor the pathogen causing the uveitis.

The Mantoux test, or TST, has to be interpreted with caution as this is subject to patient risk factors and the resultant size of the induration, and as a result false positives and false negatives may arise. For instance, an induration of ≥ 5 mm would be considered positive in high-risk patients, such as immunocompromised patients with Human Immunodeficiency Virus (HIV) or those on long-term high dose steroids, patients with end stage renal failure, those with recent contact with active cases, or those with chest x ray lesions consistent with previous TB infection. On the other hand, those with ≥ 10 mm induration are only considered positive if they are intravenous drug users, healthcare workers or those employed at high risk workplaces, paediatric patients exposed to high-risk adults, and immunocompromised patients including diabetics, long term steroid users, and those with malabsorptive state. Patients who demonstrate an induration diameter of ≥ 15 mm are deemed positive when they have no risk factors for TB. [12, 19]. Extra caution has to be taken as false positives may arise, for instance in those who have received their Bacillus Calmette-Guerin (BCG) vaccines, a particularly common practice for those residing in endemic areas such as Hong Kong. This case series highlighted a few cases for which false negatives arose using the above-mentioned guidelines. This includes 1 patient who had ≥ 5 mm induration and was considered negative as he did not have TB contact history nor CXR changes, and 1 patient who was considered negative as the induration was ≤ 5 mm, although subsequent T-SPOT.TB test yielded positive results in both patients. Although the Mantoux test is classically performed as a screening and diagnostic tool for TB, it cannot differentiate between latent and active infection, and cross-reactivity of non-tuberculous mycobacterium as well as previous BCG vaccination could result in false positives. It has also been demonstrated that in endemic areas like Hong Kong, the Mantoux test shows limited usefulness in detecting active TB disease in the elderly population. Compared to the positive rate of other diagnostic tests, chest x-ray demonstrates a rather low sensitivity so a negative chest x-ray result cannot rule out extra-pulmonary infection including ocular TB uveitis. IGRA tests, on the other hand, are advantageous in that it requires less time for generating results, and only requires one clinic visit, as opposed to the Mantoux test which requires 48–72 h of waiting time and requires the patient to come back to the clinic for reading of skin induration. The results are also relatively unaffected by previous BCG vaccine history, which is particularly useful in endemic populations. False negatives could be explained by inadequate sampling, presence of inhibitors, or ineffective lysis of cell wall and thus inadequate DNA isolation [20–22].

At present, it remains a challenge for TB infections to be diagnosed and confirmed with a single test, despite the many options for such purpose. 1 patient in our case series had TB diagnosed by Mantoux test in 1980 and treated with single agent for 6 months. In the first visit, this patient complained of sore throat and neck swelling for several month with a history of idiopathic unilateral recurrent acute anterior uveitis for two years. Chest x-ray and AFB smear were both negative, but positive for Mycobacterial infection on neck lymph node biopsy. Due to the history of recurrent uveitis and positive result on biopsy, TB was suspected and PET-CT was done to delineate the extent of systemic TB involvement and anti-TB treatment was commenced immediately. Finally, inflammation of the uvea resolved and vision improved in this case after completion of the anti-TB treatment protocol. We therefore concluded retrospectively that this patient had ocular TB, as inferred from the positive response towards anti-TB treatment.

In conclusion, TB investigation is suggested if patients have the following presentations especially in enedemic regions like Hong Kong: (1) recurrent uveitis of unknown cause; (2) chronic uveitis, especially for anterior and posterior uveitis; (3) not responding to conventional uveitic therapy; (4) immunosuppressed patients and (5) severe uvea involvement such as panuveitis or bilateral disease. We suggest that TB test should be performed routinely in uveitic patients with or without TB history or extra-ocular symptoms of TB, notably in endemic regions where TB is a differential diagnosis that has to be considered in every patient. This is because from our case series, not all of the patients had a documented or known history of latent TB disease, yet TB was the ultimate presumptive diagnosis and was eventually responsive to anti-TB therapy. Therefore, we should always have such infective differential diagnosis at the back of our minds, to detect and treat the disease in a timely manner. Taking all the pros and cons into consideration, T-SPOT. TB test and QuantiFERON gold test are useful in the diagnosis of ocular tuberculosis.

### Medical Treatment

There is still no consensus on the treatment protocol for tuberculosis uveitis, but the full treatment regime for active TB infection by the WHO is increasingly recommended. In this case series, isoniazid or rifampicin was started in the first two months, whereas other drugs including ethambutol, levofloxacin, moxifloxacin, rifampicin, pyrazinamide were added according to clinical response. Our patients were all treated in a multidisciplinary approach with respiratory physicians through directly observed treatment (DOT) and ophthalmologists monitoring the response to treatment. The treatment duration varied from 9 to 15 months, and 4 out of 9 patients received at least 12 months of anti-TB treatment, as several studies showed that prolonged anti-TB treatment can reduce uveitis recurrence [23–26]. All patients were prescribed steroids ranging from topical steroids, oral prednisolone or intravitreal dexamethasone implant in order to reduce inflammation or macular oedema. Three patients demonstrated ocular hypertension as a side effect of steroid use, and IOP lowering agent Timolol was prescribed in these patients for 4 to 15 weeks.

In our study, patient 1 (Table 2) refused to initiate anti-TB treatment and this patient experienced multiple uveitis recurrences up until 2020. When using anti-TB medications and steroids concurrently, strict monitoring is required as anti-TB drugs have mulitple side effects, as illustrated in 1 patient who experienced TB drug-induced hepatitis during treatment. 2 patients developed cystoid macular oedema, and anti-VEGF intravitreal injection was administered to reduce the oedema. Visual acuity, visual field, colour vision, urea, electrolytes and liver function should be assessed and baseline values should be obtained prior to starting treatment with ethambutol, rifampicin, isoniazid and pyrazinamide, such that any manifestations of their side effects may be picked up in a timely manner. Regular follow-ups such as OCT assessment should be performed, especially for high-risk patients including diabetics, elderly, those with renal or liver disease and chronic alcoholics.

### Treatment outcome

The visual acuity improved from 0.85 (initial) to 0.77 (final) within a mean treatment duration period of 10.63 months. The visual acuity of 60% patients was improved and 40% worsened. Among those who improved, 4 out of 6 patients were unilaterally infected and 2 out of 6 patients were bilaterally infected. This result may imply that unilateral uveitis tend to have a better visual prognosis then bilateral uveitis. In the 6 patients with improved vision, only 3 out of 6 patients had prior history of TB infection or uveitis, suggesting past history may not affect the prognosis. Moreover, we suggest that different types of uveitis have different prognoses. This study found that 100% of anterior uveitis, 75% panuveitis and 25% posterior uveitis patients had visual acuity improvement. In this case series, only 2 out of 6 patients who had complications had better final visual acuity after anti-TB treatment, so we believe that the prognosis of patients with complications may be suboptimal even after completion of anti-TB treatment.

### Limitations

Potential limitations of this study should be mentioned. A small sample size of ten patients were analyzed, as this was a single centre cross-sectional study. A wider scale study could be done in the future, incorporating cases from the entirety of the Hong Kong public and private system, to better describe the characteristics of TB uveitis in our locality.

## Conclusion

Ocular TB may manifest in various forms and could involve different parts of the eye. There is a potential for TB uveitis to evolve from unilateral to bilateral disease, and from anterior or posterior uveitis to panuveitis. Hence, both eyes should be evaluated at every follow up. In our case series, even with a small sample size, there is yet an important clinical lesson to learn – in endemic areas, or when evaluating patients from such regions, it is important to consider TB as a differential diagnosis, even though patients may not necessarily have a known or documented history of TB disease as demonstrated in this case series. In addition, in patients where there is severe involvement such as panuveitis, TB uveitis needs to be ruled out as a differential diagnosis. The presumptive diagnosis in suspicious cases requires multimodal investigations where a negative chest x-ray is not useful in ruling out ocular TB infections. It is important to conduct tuberculosis investigations in a timely manner to avoid prolonging the uveitis, which could lead to poor visual outcome. Collaboration between TB clinic and ophthalmic clinic in monitoring disease progression in patients is beneficial to the their prognoses and visual outcomes.

## Data Availability

The authors agree to make all materials, data and associated protocols promptly available to readers without undue qualifications in material transfer agreements.

## Abbreviations

BCG: bacillus Calmette–Guerin
CDC: Centers for Disease Control and Prevention
CXR: chest x-ray
CMV: cytomegalovirus
DOT: directly observed treatment
EBV: Epstein Barr virus
HSV: herpes simplex virus
HIV: Human Immunodeficiency Virus
IRB: Institutional Review Board
IGRA: interferon-γ releasing assay
IOP: intraocular pressure
OCT: optical coherence tomography
PCR: Polymerase Chain Reaction
PPD: Purified Protein Derivative
TB: tuberculosis
TST: tuberculin skin test
VZV: Varicella-Zoster Virus
WHO: World Health Organisation
